# Determining the direction of prediction of the association between parasympathetic dysregulation and exhaustion symptoms

**DOI:** 10.1038/s41598-022-14743-4

**Published:** 2022-06-23

**Authors:** Magdalena K. Wekenborg, Andreas Schwerdtfeger, Nicole Rothe, Marlene Penz, Andreas Walther, Clemens Kirschbaum, Julian F. Thayer, Ralf A. Wittling, LaBarron K. Hill

**Affiliations:** 1 Department of Biological Psychology, TU Dresden, Dresden, Germany; 2 Else Kröner Fresenius Center for Digital Health, University Hospital and Medical Faculty Carl Gustav Carus, TU Dresden, Dresden, Germany; 3 Institute of Psychology, University of Graz, Graz, Austria; 4 Institute of Education and Psychology, Johannes Kepler University, Linz, Austria; 5 Clinical Psychology and Psychotherapy, University of Zurich, Zurich, Switzerland; 6 Department of Psychological Science, School of Social Ecology, University of California, Irvine, CA 92697 USA; 7 ZNF-Center for Neuroscience Research NPO, Trier, Germany; 8 Department of Psychiatry and Behavioral Sciences, Duke University Medical Center, Durham, NC USA

**Keywords:** Physiology, Psychology, Biomarkers

## Abstract

Stress-related exhaustion symptoms have a high prevalence which is only likely to increase further in the near future. Understanding the physiological underpinnings of exhaustion has important implications for accurate diagnosis and the development of effective prevention and intervention programs. Given its integrative role in stress-regulation, the parasympathetic branch of the autonomic nervous systems has been a valid starting point in the exploration of the physiological mechanisms behind exhaustion. The aim of the present study was to examine the directionality and specificity of the association between exhaustion symptoms and vagally-mediated heart rate variability (vmHRV), a relatively pure measure of parasympathetic tone. Exhaustion symptoms and vmHRV were measured at four annually assessment waves (2015–2018) of the Dresden Burnout Study. A total sample of N = 378 participants who attended at least two of the four annual biomarker measurements were included in the present analyses. Cross-lagged multi-level panel modelling adjusting for various covariates (e.g., age, sex, BMI) revealed that vmHRV was meaningfully predictive of exhaustion symptoms and not vice versa. In addition, these effects were specific for exhaustion symptoms as no effect was shown for the other burnout sub-dimensions, or for depressive symptoms. Our findings indicate a clear link between exhaustion symptoms and vmHRV which may hold great potential for both enhancing the diagnosis and treatment of exhaustion symptoms.

## Introduction

Exhaustion symptoms, defined as the depletion of energetic resources as a result of prolonged exposure to chronic stress^1^, exhibit a high prevalence worldwide^2–4^. This trend is especially alarming given the severe repercussions of exhaustion symptoms for the individual (i.e., enhanced all-cause mortality^5^), as well as its considerable financial burden for society due to exhaustion associated work-disability^6^ and future hospital admissions^7^. Exhaustion symptoms have been most extensively studied within the theoretical framework of burnout. Most researchers in the field, as well as the ICD-11^8^ characterize burnout based on the definition introduced by Maslach, et al.^9^. Maslach, et al.^9^ defined burnout as a consequence of chronic stress with its core symptom exhaustion, accompanied by cynicism (i.e., a negative attitude towards clients and co-workers), and reduced personal accomplishment (i.e., reduced perceived productivity). The severe consequences underscore the substantial importance of studying plausible psychophysiological pathways of exhaustion symptoms for two main goals: (1) identifying biomarkers that might improve early diagnosis of exhaustion symptoms, and (2) developing effective prevention and treatment strategies based on a more profound understanding of the link between chronic stress exposure and the development of exhaustion symptoms.

As detailed in the model of neurovisceral integration, the appraisal of acute stress causes, via direct influence of cortical and limbic circuits on the autonomic nervous system (ANS), a temporal dominance of the sympathetic nervous system (SNS) over the parasympathetic nervous system (PNS), which is, at least from an evolutionary perspective, adaptive as it provides energy mobilization to overcome (i.e., evaluate, fight, flee) inciting environmental challenge. However, in case of enduring experiences of stress (i.e., chronic stress) this initially adaptive dynamic shifts to an established state of dysregulation, wherein the SNS influence is continuously dominant and PNS activity is diminished and/or ineffective, which poses excessive energy demands on the body^10^. Importantly, PNS activity can be indexed by vagally-mediated heart rate variability (vmHRV). HRV is thereby defined as differences in the time intervals between sequential heart beats, which result from the interplay of the SNS and the PNS. Due to differences in neurotransmitter signalling, only the PNS (via the vagus nerve) is able to modulate the heart rate on a time scale of milliseconds^11–13^, making high-frequency changes in HRV a relatively pure measure of vagal function.

Over time, this PNS dysregulation results in energy depletion, making it impossible for the individual to meet environmental demands^14^. And indeed, previous findings indicate that chronic stress leads to vagal withdrawal as indexed by reduced vmHRV^15^.

We were the first to explicitly show that the exhaustion dimension of burnout is most reliably associated with a dysregulation of PNS^16,17^, which is in line with previous studies outside the burnout framework^18,19^. There is, however, a lack of studies investigating the causal direction between exhaustion symptoms and vmHRV. Most prior studies on burnout symptoms and vagal function were cross-sectional, limiting their ability to assess the temporal association^20,21^. Our findings indicate a bidirectional relationship, as both exhaustion symptoms predicted low vmHRV, and low vmHRV predicted enhanced exhaustion symptoms over a 1-year period^17^. The only other two studies examining the longitudinal associations of burnout symptoms and vmHRV of which the authors are aware, were both conducted in Chinese patients after acute coronary syndrome^22,23^. Zhang et al.^22^ and Shi et al.^23^ could demonstrate that burnout symptoms, operationalized using the Copenhagen Burnout Inventory^24^, a measure with an explicit focus on the exhaustion dimension of burnout, predicted reduced vmHRV over a 1-year period. Since burnout symptoms were assessed at baseline only, no conclusion can be made about the directionality of their association. In addition, longer time periods are needed to draw firm conclusions about directionality, as to the authors knowledge all previous longitudinal studies are limited to a 1-year period.

In summary, there are indications of exhaustion-associated modulations in vagal function but studies investigating the causal link are lacking. In addition, there is some uncertainty regarding the specificity of exhaustion-associated modulations in vmHRV with respect to other dimensions of burnout as well as depressive symptoms. Previous research has primarily focused on associations between low vmHRV and diagnostic categories (i.e., depressed vs. not depressed), or examined composite symptom scores. This research yielded inconclusive results (review^25,26^). Previous work from our group indicates that exhaustion symptoms are the predominant driver of the association with reduced vagal function irrespective of the specific classification/diagnostic category employed (i.e., depression vs. burnout)^16^. This is consistent with other previous work demonstrating that somatic, but not cognitive-affective symptoms are mainly responsible for associations between depression and vmHRV^26^. Following this line of reasoning, the observation of significant associations of vmHRV with burnout or depression may depend largely on the chosen operationalization.

Utilizing data collected across the four annual biomarker sampling points of the Dresden Burnout Study (2015–2018), the present study further investigated the directionality and specificity of the association between exhaustion symptoms and vmHRV. We employ a multilevel cross-lagged panel design^27^ which maximizes the utility of our large sample size and extensive observation period. Based on previous findings^16,17^, we hypothesized that the cross-temporal association between vmHRV and exhaustion symptoms would be distinct from the other burnout sub-dimensions, as well as depressive symptoms. No hypothesis was made regarding the directionality of this association.

## Methods

### Study population

The present study included participants from the ongoing Dresden Burnout Study. Recruitment strategies and design of the Dresden Burnout Study are described in detail elsewhere^28^. Briefly, the Dresden Burnout Study is a large-scale longitudinal study designed to systematically assess health-related risk and protective factors of burnout symptoms. Participants were recruited from across Germany via public media, as well as via the civil register of the city of Dresden. In order to ensure a heterogeneous sample composition, the only inclusion criteria were an age between 18 and 68 years, as well as German language skills. The Dresden Burnout Study includes an online assessment of a range of demographic, psychological, and general health related factors via the official study homepage (www.dresdner-burnout-studie.de). In addition, since its start in 2015, participants with residence in Dresden and within a 60 km radius around the city are annually invited for biomarker sampling including the collection of heart rate data as well as blood and hair samples.

The present study included those participants with complete heart rate data and relevant sociodemographic and health related factors for at least two of the four biomarker sampling points between 2015 and 2018 resulting in a sample of N = 392. After exclusion of outliers (n = 5 individuals with vmHRV values M < 3SD; n = 5 individuals with BMI > 45), a final sample of N = 378 participants were included in the present analyses. Detailed sample characteristics of those individuals included in the present analyses at each biomarker sampling point are depicted in Table 1.

**Table 1:** Sample characteristics for those individuals included in the present analyses (Unless otherwise stated, number in brackets are standard deviations).

	Biomarker sampling 1 (Sept.–Oct. 2015)	Biomarker sampling 2 (Oct. 2016–Feb. 2017)	Biomarker sampling 3 (Oct.–Dec. 2017)	Biomarker sampling 4 (Oct.–Dec. 2018)
n	391	471	401	469
Age (years)	41.79 (11.17)	40.57 (12.02)	42.95 (11.68)	43.15 (11.68)
Sex (female)	259 (66.2%)	321 (68.2%)	290 (72.3%)	330 (70.4%)
**Health related variables**				
BMI (kg/m^2^)	25.25 (4.54)	24.53 (4.39)	25.58 (4.87)	25.62 (4.90)
Smokers (ys)	49 (12.5%)	68 (14.4%)	55 (13.7%)	59 (12.6%)
Alcohol consumption (ys)	341 (87.2%)	430 (91.3%)	367 (91.5%)	429 (91.5%)
Cardiovascular disease (ys)	79 (20.2%)	76 (16.1%)	74 (18.5%)	89 (19.0%)
MBI total score	2.19 (1.16)	2.14 (1.07)	2.19 (1.10)	2.21 (1.11)
Emotional exhaustion	2.79 (1.56)	2.75 (1.50)	2.83 (1.48)	2.85 (1.50)
Cynicism	1.98 (1.50)	2.00 (1.39)	2.04 (1.49)	2.06 (1.45)
Reduced personal accomplishment	1.61 (1.12)	1.48 (0.97)	1.48 (1.03)	1.50 (0.97)
PHQ-9 total score	8.17 (5.20)	7.83 (5.20)	7.65 (4.88)	7.52 (5.01)
PHQ-9 cog	3.98 (2.95)	3.69 (2.88)	3.61 (2.76)	3.50 (2.75)
PHQ-9 som	4.19 (2.62)	4.14 (2.71)	4.04 (2.50)	4.02 (2.65)
RMSSD	35.32 (21.84)	38.25 (22.83)	36.42 (23.58)	38.75 (23.57)
HF-HRV	517.18 (841.82)	563.33 (806.62)	531.15 (849.30)	604.62 (913.12)

*BMI* body mass index, *Cardiovascular Disease* self-reported hypertension and/or cardiac arrhythmias, *HF-HRV* high frequency heart rate variability, *MBI* Maslach Burnout Inventory, *PHQ-9* Patient Health Questionnaire sum-score, *PHQ-9 cog* Patient Health Questionnaire 9—cognitive factor, *PHQ-9 som* Patient Health Questionnaire 9—somatic factor, *RMSSD* root mean square of successive difference between heart beats, *ys* number of participants who answered the respective question with yes.

HRV, as well as questionnaire data collected at the first and the second biomarker sampling points has already been published^10,11^. HRV data collected at the third and fourth biomarker sampling point has never been published before.

All participants gave written informed consent. The Dresden Burnout Study has been developed in accordance with the Declaration of Helsinki, and has been approved by the local ethics committee of the Technical University Dresden. All participants received a monetary reward of 15 € per biomarker sampling time point.

### Protocol

Each biomarker sampling point followed a standardized procedure which is described below. Within one week before the biomarker sampling, participants completed an online questionnaire via the study homepage, assessing burnout and depressive symptoms, as well as sociodemographic and health related factors. Laboratory sessions lasted approximately 50 min and were conducted between 7 a.m. and 7 p.m. (for economic reasons, ECG data could not be collected from participants at the same time of day at each measurement point). Participants were told to refrain from drinking alcohol and caffeine, smoking, and strenuous physical activity during the day of the laboratory sessions. On arrival, participants read and signed informed-consent forms and were provided with a heart rate device which they wore during the whole biomarker sampling procedure as follows: blood draw, hair sample collection, and a 335-s heart rate recording in a seated position (resting condition) with spontaneous breathing. Heart rate recording was initiated following an approximately 60-s stabilization period on a seated position. During this stabilization period, participants were instructed to remain seated quietly for the next six minutes, as unnecessary movement can distort the heart rate. In order to avoid possible further distortions of vmHRV^29^, no instructions for cognitive-demanding tasks were given.

A seated resting condition seems especially suited to examine burnout associated changes in autonomic function as this experimental setting has previously been shown to enable assessment of HRV as a trait-like marker of vagal function^30^.

### Self-report measures

The following covariates that have previously been shown to influence cardiac vagal tone were assessed: age, sex, alcohol consumption (yes/no), smoking (yes/no), physician diagnosed cardiovascular disease (hypertension and/or cardiac arrhythmias; yes/no) via self-report. Body mass index (BMI) was calculated based on participants’ self-reported weight and height.

For consideration within the scope of sensitivity analyses, cardiovascular risk factors (also via self-report) were assessed (i.e., diabetes [yes/no], high cholesterol [yes/no]).

Exhaustion symptoms were measured using the emotional exhaustion sub-scale German version (MBI-GS-D^31^) of the Maslach Burnout Inventory General Survey (MBI-GS^1^). In order to be able to explore the role of overall burnout symptoms, as well as the other two subscales from the MBI (i.e., cynicism, reduced personal accomplishment), participants rated all 16 items of the MBI on a seven-point Likert scale (0 = never; 6 = daily). All MBI sub-scales showed good reliabilities over the four measurement time points indicated by Cronbach’s Alpha values between 0.84 and 0.92 (emotional exhaustion: 0.92; cynicism: 0.84–0.87; reduced personal accomplishment: 0.84–0.86). The weighted MBI total score (0.4 × EE + 0.3 × cynicism + 0.3 × reduced personal accomplishment) was calculated as suggested by Kalimo et al.^32^. The MBI total score and its three subscales were considered as continuous variables and, due to high intercorrelations, analysed separately.

Depressive symptoms were assessed with the German version (PHQ9-D^33^) of the Patient Health Questionnaire (PHQ-9^34^). The PHQ-9 consists of nine items which are scored on a 4-point ranking scale (0 = not at all; 3 = nearly all day). The items quantify the frequency of each of the nine diagnostic criteria for a depressive disorder over the last two weeks of the Diagnostic and Statistical Manual of Mental Disorders^35^ and can be summed up to a continuous variable (PHQ-9 sum score), with higher scores representing higher severity of depressive symptoms. As previous research indicates a special role for somatic depressive symptoms compared to cognitive depressive symptoms^16,26^, separate somatic and cognitive symptom scores were derived within the PHQ-9. Accordingly to De Jonge et al.^36^ the items assessing exhaustion, sleeping problems, changes in appetite, and psychomotor agitation were summed to form the PHQ-9 somatic factor (Cronbach’s Alpha for the four measurement time points: 0.74–0.77), whereas the items lack of interest, depressed mood, negative feelings about self, concentration problems, and suicidal intention were subsumed in the PHQ-9 cognitive factor (Cronbach’s Alpha for the four measurement time points: 0.79–0.82).

### VmHRV measurements

Inter-beat intervals (IBI) were assessed using a wireless chest transmitter and a wrist monitor recorder (Polar RS800CX system; Polar Electro OY, Kempele, Finland) with a sampling frequency of 1000 Hz throughout the biomarker sampling procedure. Of the complete IBI timeline of the respective sampling point, only a 335-s period (about 5.5 min) of the seated resting condition was analyzed in the present study to capture an index of tonic vmHRV. IBIs were available for: (1) n = 434 invidiuals at T1 (Sept.–Oct. 2015) n = 434); (2) n = 527 invidividuals at T2 (Oct. 2016–Feb. 2017); n = 438 individuals at T3 (Oct.–Dec. 2017); n = 511 at T4 (Oct.–Dec. 2018).

The data of each sampling point were then transferred to the Polar Precision Performance Software (Polar Electro OY, Kempele, Finland) and exported as the raw IBI data for further analysis. The ECG raw data were artefact corrected and the most frequently used primarily vmHRV measures (i.e., the time-domiane measure root mean sum of squares of successive differences [RMSSD]; frequency-domain measure high-frequeny HRV [HF-HRV; frequency band, 0.15–0.4 Hz]) were calculated by a third party, namely the NEUROCOR Ltd. & Co. KG (Trier, Germany), according to the guidelines of the Task Force^37^ using the NEUROCOR precision HRV-Algorithm. More precisely, first, detection of R-spikes was performed using a modified Pan-Thompkins-Algorithm. Second, milliseconds based RR intervals of the segment were articact checked automatically by the „NEUROCOR precisionHRV-Algorithm “-based on the European Patent “EP2745770B1 Method and device for determining the variability of a creature’s heart rate”. During the correction process the RR data was segmented in continuous intervals of 335 s. Milliseconds based RR intervals of the segment were artifact checked automatically. The algorithm marks all RR intervals outside physiological limits or values that are not suitable for a HRV analysis. This includes RR intervals < 400 ms, RR intervals > 2000 ms and RR intervals whose moving average over 5 intervals exceeds an age-dependent threshold. In addition, RR intervals are marked which are recognized as statistical outliers. The correction of the artifacts takes place without a violation of the alignment of the phases and without a change of the total signal time. To this the affected areas are not cut out, but replaced by spline interpolated RR times of the same length. For larger artifact phases, the distribution of the RR times is adapted to the RR time dynamics before and after the defect. The number of RR intervals replaced was counted and converted to a percentage based on the total number of all heartbeats per 335 s segment. Only segments with an artifact load ≤ 5% were included in the calculation of the HRV analysis. This criterion was met for: (1) n = 413 invidiuals at T1 (Sept.–Oct. 2015); (2) n = 503 invidividuals at T2 (Oct. 2016–Feb. 2017); n = 423 individuals at T3 (Oct.–Dec. 2017); n = 497 at T4 (Oct.–Dec. 2018). Third, HRV measures were calculated. RMSSD was used to operationalize vmHRV because it is an approved short-term measure of HRV reflecting vagal cardiac influence and its robustness against breathing patterns^37,38^. RMSSD values at rest were not normally distributed; thus, log transformations were applied to reduce skewness. To examine potential differences between different vmHRV operationalizations, we conducted the main analyses with lnRMSSD and lnHF-HRV (also log-transformed due to skeweness).

### Statistical analyses

We used multilevel cross-lagged panel models to assess bidirectional pathways linking vmHRV and exhaustion symptoms across time. Such analyses allow for a clear investigation of the directionality of associations between constructs by allowing for the simultaneous estimation of relations whereby each variable at the first measurement time point is allowed to predict variables at the next measurement time point (cross-lagged component). These relations are also estimated while controlling for the longitudinal stability of each construct (autoregressive component) and calculating the residualized covariance, so that results reflect the effects of each construct measured at the first time point on increases or decreases in other constructs relative to the baseline level, thereby controlling for the other paths. The parameters in all models were estimated using the robust maximum-likelihood estimation method. Models were calculated using Bayesian modelling with non-informative priors. Models were calculated with random slopes (measurement time) and random intercepts for each participant.

Available data from all four biomarker sampling points were included in the analyses. Since not all study participants took part in all measurement time points, only the available time intervals between the measurement time points served as the basis for the analyses. Therefore, the longest time lag included in the analyses was three years, the shortest one year.

Our primary analysis focused on exhaustion symptoms longitudinally predicting vmHRV and conversely (vmHRV longitudinally predicting exhaustion symptoms). The comparison of the magnitude of the associations between these two pathways provides information on the temporal sequence, or directionality, between the two variables. More precisely, autoregressive and cross-lagged paths were modelled for exhaustion symptoms and lnRMSSD. In addition, age, sex, BMI, the PHQ-9 somatic and cognitive factors, as well as smoking, alcohol consumption, and self-reported cardiovascular disease at the respective measurement time point were entered into the model as covariates. In order to ensure a comprehensive assessment of vmHRV, we conducted the same multilevel cross-lagged panel model for lnHF-HRV too.

In a second step, we evaluated the specificity of potentially revealed associations with lnRMSSD with respect to burnout symptoms by running the same multilevel cross-lagged panel models, but replacing exhaustion symptoms with the respective burnout score. Thereby, the MBI total score, cynicism and reduced personal accomplishment were examined separately in order to avoid problems with multicollinearity, and to reduce model complexity. Each of these models included lnRMSSD as the corresponding core variable, and age, sex, BMI, the PHQ-9 somatic and cognitive factors, smoking, alcohol consumption, and cardiovascular disease at the respective measurement time point as covariates.

In a third step, in order to examine the specificity of exhaustion associated modulations in vmHRV with respect to depressive symptoms, two separate multilevel cross-lagged panel models were calculated for the PHQ-9 cognitive factor and the PHQ-9 somatic factor. As the results were virtually identical for the PHQ-9 total score and its sub-scores, results for the PHQ-9 total score are not depicted here. The different PHQ-9 scores were examined separately in in order to avoid problems with multicollinearity, and to reduce model complexity. Each of these models included lnRMSSD as the corresponding core variable, and age, sex, BMI, the PHQ-9 somatic and cognitive factor, smoking, alcohol consumption, and cardiovascular diseases at the respective measurement time point as covariates.

Additionally, sensitivity analyses were performed, which included all cardiovascular diseases (i.e., hypertension, cardiac arrhythmias) and cardiovascular risk factors (i.e., diabetes, high cholesterol) within the multilevel cross-lagged panel models described in the steps one to three.

All statistical analyses were conducted using *R*^39^ package *brms* (Version: 2.15.0) using non-informative priors. Parameters of multilevel cross-lagged panel models were considered meaningful (i.e., the revealed effect is reliable), if the associated CI does not contain zero.

## Results

**Figure 1:**
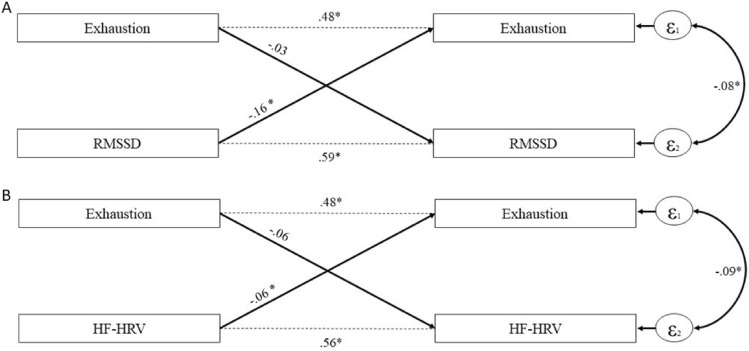
Cross-lagged associations between exhaustion symptoms and vagally-mediated heart rate variability. Illustration of the cross-lagged association between exhaustion symptoms and (**A**) RMSSD (root mean square of successive difference between heart beats, logarithmized), as well as (**B**) HF-HRV (high frequency heart rate variability, logarithmized), adjusted for sex, age, Body Mass Index, Patient Health Questionnaire 9—cognitive factor, Patient Health Questionnaire 9—somatic factor, smoking, alcohol consumption, and cardiovascular diseases. The numbers indicate unstandardized effect estimates which refer to logarithmized vmHRV values. T1 = first attended measurement time point; T2 = measurement time point with the largest time-lag to T1. *CI does not include zero.

We tested the magnitude and directionality of the association between exhaustion symptoms and lnRMSSD via a multilevel cross-lagged panel model with covariates. Results are depicted in Table 2 and Fig. 1. We found that higher lnRMSSD meaningfully predicted lower exhaustion symptoms (b = − 0.16; 95% CI [− 0.29, − 0.04]). In contrast, the converse longitudinal association (lower exhaustion symptoms predicting higher lnRMSSD) was not meaningful (b = − 0.03; 95% CI [− 0.06, 0.00]), indicating an uni-directional association between lnRMSSD to exhaustion symptoms. The results were virtually identical for lnHF-HRV (Table 3): Mirroring the results of lnRMSSD, higher lnHF-HRV also meaningfully predicted lower exhaustion symptoms (b = − 0.06; 95% CI [− 0.12, − 0.00]). Also in line with the results for lnRMSSD, exhaustion symptoms did not meaningfully predict lnHF-HRV (b = − 0.06; 95% CI [− 0.12, 0.01]), emphasizing the importance of vmHRV predicting exhaustion symptoms.

**Table 2:** Path coefficients of cross-lagged panel models on exhaustion symptoms and lnRMSSD with covariates.

	lnRMSSD–Exhaustion model			
	lnRMSSD		Exhaustion	
	Estimate	CI	Estimate	CI
Stability paths (T1–T2)	0.59*	0.52, 0.65	0.48*	0.42, 0.53
Cross-lagged effects (T1–T2)	Exhaustion → lnRMSSD		lnRMSSD → Exhaustion	
	− 0.03	− 0.06, 0.00	− 0.16*	− 0.29, − 0.04
**Effects of covariates (T1)**				
Sex	0.02	− 0.05, 0.10	< 0.01	− 0.14, 0.14
Age	− 0.01*	− 0.01, − 0.01	< 0.01	− 0.00, 0.01
BMI	< 0.01	− 0.01, 0.00	< 0.01	− 0.02, 0.01
PHQ-9 som	0.01	− 0.01, 0.03	0.10*	0.07, 0.14
PHQ-9 cog	− 0.02	− 0.03, 0.00	0.15*	0.12, 0.19
Smoking	− 0.12*	− 0.22, − 0.02	0.17	− 0.03, 0.37
Alcohol consumption	− 0.01	− 0.13, 0.11	0.07	− 0.16, 0.30
Cardiovascular disease	< 0.01	− 0.10, − 0.10	− 0.06	− 0.25, 0.12

Sex is coded 0 = male, 1 = female; BMI = body mass index; Cardiovascular Disease = self-reported hypertension and/or cardiac arrhythmias; CI = confidence interval; PHQ-9 cog = Patient Health Questionnaire 9—cognitive factor; PHQ-9 som = Patient Health Questionnaire 9—somatic factor; lnRMSSD = root mean square of successive difference between heart beats, logarithmized; T1 = first attended measurement time point; T2 = measurement time point with the largest time-lag to T1.

*CI does not include zero.

**Table 3:** Path coefficients of cross-lagged panel models on exhaustion symptoms and lnHF-HRV with covariates.

	lnHF-HRV–Exhaustion model			
	lnHF-HRV		Exhaustion	
	Estimate	CI	Estimate	CI
Stability paths (T1–T2)	0.56*	0.50, 0.63	0.48*	0.42, 0.53
Cross-lagged effects (T1–T2)	Exhaustion → lnHF-HRV		lnHF-HRV → Exhaustion	
	− 0.06	− 0.12, 0.01	− 0.06*	− 0.12, − 0.00
**Effects of covariates (T1)**				
Sex	0.20*	0.03, 0.37	< 0.01	− 0.15, 0.15
Age	− 0.02*	− 0.03, − 0.02	< 0.01	− 0.00, 0.01
BMI	< 0.01	− 0.02, 0.01	< 0.01	− 0.02, 0.01
PHQ-9 som	0.02	− 0.02, 0.07	0.10*	0.07, 0.14
PHQ-9 cog	− 0.05*	− 0.09, − 0.00	0.15*	0.12, 0.19
Smoking	− 0.17	− 0.40, 0.06	0.17	− 0.04, 0.37
Alcohol consumption	− 0.08	− 0.36, 0.20	0.07	− 0.15, 0.29
Cardiovascular disease	− 0.02	− 0.24, 0.18	− 0.05	− 0.23, 0.13

Sex is coded 0 = male, 1 = female; BMI = body mass index; Cardiovascular Disease = self-reported hypertension and/or cardiac arrhythmias; CI = confidence interval; lnHF-HRV = high frequency heart rate variability, logarithmized; PHQ-9 cog = Patient Health Questionnaire 9—cognitive factor; PHQ-9 som = Patient Health Questionnaire 9—somatic factor; T1 = first attended measurement time point; T2 = measurement time point with the largest time-lag to T1.

*CI does not include zero.

### Specificity of the revealed effects with respect to other burnout symptoms

Results of multilevel cross-lagged panel models on the association between lnRMSSD and other burnout symptoms support the specificity of the revealed effects for exhaustion symptoms as none of the other burnout scores (MBI total score, cynicism, reduced personal accomplishment) exhibited meaningful paths with lnRMSSD (Table 4).

**Table 4:** Path coefficients of cross-lagged panel models on burnout scores and lnRMSSD with covariates.

	lnRMSSD–MBI model				lnRMSSD–CY model				lnRMSSD–PEr model			
	lnRMSSD		MBI		lnRMSSD		CY		lnRMSSD		PEr	
	Estimate	CI	Estimate	CI	Estimate	CI	Estimate	CI	Estimate	CI	Estimate	CI
Stability paths (T1–T2)	0.60*	0.53, 0.66	0.53*	0.47, 0.58	0.60*	0.53, 0.66	0.52*	0.46, 0.58	0.60*	0.53, 0.66	0.51*	0.45, 0.57
Cross-lagged effects (T1–T2)	MBI → lnRMSSD		lnRMSSD → MBI		CY → lnRMSSD		lnRMSSD → CY		Per → lnRMSSD		lnRMSSD → Per	
	− 0.01	− 0.05, 0.03	− 0.08	− 0.16, 0.00	0.01	− 0.02, 0.03	− 0.10	− 0.24, 0.05	0.02	− 0.02, 0.05	0.03	− 0.08, 0.13
**Effects of covariates (T1)**												
Sex	0.02	− 0.06, 0.10	− 0.04	− 0.14, 0.06	0.02	− 0.06, 0.10	− 0.09	− 0.26, 0.09	0.02	− 0.06, 0.09	− 0.02	− 0.15, 0.10
Age	− 0.01*	− 0.01, − 0.01	< 0.01	− 0.00, 0.01	− 0.01*	− 0.01, − 0.01	< 0.01	− 0.01, 0.01	− 0.01*	− 0.01, − 0.01	< 0.01	− 0.00, 0.01
BMI	< 0.01	− 0.01, 0.00	< 0.01	− 0.01, 0.01	< 0.01	− 0.01, 0.00	0.02	− 0.00, 0.03	< 0.01	− 0.01, 0.01	− 0.01	− 0.02, 0.00
PHQ-9 som	0.01	− 0.01, 0.03	0.04*	0.02, 0.07	0.01	− 0.01, 0.03	0.01	− 0.03, 0.06	0.01	− 0.01, 0.03	0.01	− 0.03, 0.04
PHQ-9 cog	− 0.02*	− 0.04, − 0.00	0.13*	0.11, 0.16	− 0.02*	− 0.04, − 0.01	0.16*	0.12, 0.20	− 0.02*	− 0.04, − 0.01	0.09*	0.06, 0.12
Smoking	− 0.12*	− 0.22, − 0.01	0.11	− 0.03, 0.24	− 0.12*	− 0.22, − 0.02	0.08	− 0.15, 0.32	− 0.12*	− 0.22, − 0.01	0.05	− 0.10, 0.22
Alcohol consumption	< 0.01	− − 0.13, 0.12	0.06	− 0.09, 0.22	< 0.01	− 0.12, 0.11	0.11	− 0.17, 0.38	< 0.01	− 0.12, 0.12	< 0.01	− 0.19, 0.20
Cardiovascular disease	< 0.01	− 0.09, 0.10	− 0.01	− 0.14, 0.12	0.01	− 0.09, 0.10	− 0.04	− 0.27, 0.18	0.01	− 0.10, 0.10	0.07	− 0.09, 0.22

Sex is coded 0 = male, 1 = female; BMI = body mass index; Cardiovascular Disease = self-reported hypertension and/or cardiac arrhythmias; CI = confidence interval; CY = Maslach Burnout Inventory – cynicism sub score; MBI = Maslach Burnout Inventory GS – total score; Per = Maslach Burnout Inventory – reduced personal accomplishment sub score; PHQ-9 cog = Patient Health Questionnaire 9—cognitive factor; PHQ-9 som = Patient Health Questionnaire 9—somatic factor; lnRMSSD = root mean square of successive difference between heart beats, logarithmized; T1 = first attended measurement time point; T2 = measurement time point with the largest time-lag to T1.

*CI does not include zero.

### Specificity of the effects with respect to depressive symptoms

Results of multilevel cross-lagged panel models on the association between lnRMSSD and the two depressive symptom factors (i.e., PHQ-9 somatic factor; PHQ-9 cognitive factor) are depicted in Table 5. Analyses revealed no meaningful cross-lagged paths between the two depressive symptom factors with lnRMSSD, indicating exhaustion-specificity with respect to depressive symptoms.

**Table 5:** Path coefficients of cross-lagged panel models on PHQ-9 factors and lnRMSSD with covariates.

	**RMSSD-PHQ-9 som model**				**RMSSD-PHQ-9 cog model**			
	lnRMSSD		PHQ-9 som		lnRMSSD		PHQ-9 cog	
	Estimate	CI	Estimate	CI	Estimate	CI	Estimate	CI
Stability paths (T1–T2)	0.60*	0.53, 0.66	0.62*	0.56, 0.68	0.60*	0.53, 0.66	0.67*	0.61, 0.72
Cross-lagged effects (T1–T2)	PHQ-9 som → lnRMSSD		lnRMSSD → PHQ-9 som		PHQ-9 cog → lnRMSSD		lnRMSSD → PHQ-9 cog	
	− 0.01	− 0.02, 0.01	− 0.19	− 0.49, 0.09	− 0.01	− 0.02, 0.01	0.01	− 0.27, 0.30
**Effects of covariates (T1)**								
Sex	0.03	− 0.05, 0.11	0.09	− 0.23, 0.42	0.03	− 0.05, 0.10	− 0.11	− 0.44, 0.22
Age	− 0.01*	− 0.01, − 0.01	− 0.01	− 0.02, 0.01	− 0.01*	− 0.01, − 0.01	− 0.02*	− 0.03, − 0.00
BMI	< 0.01	− 0.01, 0.00	0.02	− 0.02, 0.06	< 0.01	− 0.01, 0.00	0.03	− 0.01, 0.06
Smoking	− 0.13*	− 0.23, − 0.02	− 0.06	− 0.50, 0.37	− 0.13*	− 0.23, − 0.03	0.17	− 0.28, 0.62
Alcohol consumption	< 0.01	− 0.12, 0.12	− 0.65*	− 1.16, − 0.14	< 0.01	− 0.12, 0.11	− 0.19	− 0.73, 0.36
Cardiovascular disease	< 0.01	− 0.09, 0.10	− 0.06	− 0.49, 0.35	< 0.01	− 0.10, 0.10	0.15	− 0.28, 0.58

Sex is coded 0 = male, 1 = female; BMI = body mass index; Cardiovascular Disease = self-reported hypertension and/or cardiac arrhythmias; CI = confidence interval; PHQ-9 cog = Patient Health Questionnaire 9—cognitive factor; PHQ-9 som = Patient Health Questionnaire 9—somatic factor; lnRMSSD = root mean square of successive difference between heart beats, logarithmized; T1 = first attended measurement time point; T2 = measurement time point with the largest time-lag to T1.

*CI does not include zero.

### Sensitivity analyses

In order to further examine the role of cardiovascular disease and risk factors within the models described above (i.e., hypertension, cardiac arrhythmias, high cholesterol, diabetes) all models were additionally conducted including these variables as covariates. Detailed description of the results of these sensitivity analyses are depicted in the appendix (Supplementary Tables 1–4). Shortly summarized, the previously revealed results were robust with respect to lnRMSSD, as higher lnRMSSD still predicted lower exhaustion symptoms after inclusion of the cardiovascular risk factors (b = − 0.15; 95% CI [− 0.28, − 0.02]), whereby the converse longitudinal association was meaningful, too (b = − 0.03; 95% CI [− 0.06, − 0.00]; Supplementary Table 1). In contrast, no meaningful results were revealed for lnHF-HRV within these models: neither did lnHF-HRV meaningfully predict lower exhaustion symptoms (b = − 0.06; 95% CI [− 0.12, 0.01]), nor did exhaustion symptoms meaningfully predict lnHF-HRV (b = − 0.06; 95% CI [− 0.12, 0.00]; Supplementary Table 2).

No differences in results were revealed with respect to the other burnout (Supplementary Table 3) and depressive symptoms (Supplementary Table 4).

## Discussion

Exhaustion symptoms and their potentially severe repercussions for the individual and society have grown in relevance as a consequence of increased chronic stress levels at work and beyond^40^. This development underlines the importance of understanding the psychophysiological aetiology of exhaustion in order to develop powerful prevention and intervention programs.

Our study is the first to seemingly confirm the directionality of the association between exhaustion symptoms and reduced vagal function, operationalized by vmHRV measures from four annual waves of the Dresden Burnout Study. Notably, our findings indicate that in participants with multiple observations during the four annual waves of the Dresden Burnout Study, higher vmHRV predicts reduced exhaustion symptoms, and that this association is not recursive. In addition, our study provides evidence on the specificity of this relation, as neither of the other burnout dimensions (i.e., cynicism, reduced personal accomplishment) nor depressive symptoms showed comparable cross-temporal associations with vmHRV.

Our finding on the unidirectional association between vmHRV and exhaustion symptoms expands previous cross-sectional findings of a negative association between these two constructs within the burnout framework^16^, and beyond^18,19^. The finding that vmHRV predicted exhaustion symptoms, and not vice versa, contradicts the existing longitudinal studies of this association. Our own previous research indicated a bidirectional relationship^17^, whereas Zhang et al.^22^ and Shi et al.^23^ showed that vmHRV was predicted by a burnout measure with an explicit focus on exhaustion symptoms. Several explanations for these divergent findings are plausible. First, as Zhang et al.^22^ and Shi et al.^23^ did not assess vmHRV at baseline, they were unable to test the reverse directionality. In addition, all three studies only examined a 1-year time-interval. A major strength of the present study is the inclusion of exhaustion and ANS data spanning three years which enables a more specific disentangling of causal mechanisms. Another advantage of the present study is the usage of a multilevel cross-lagged panel design (an especially robust statistical tool which uses all available data and observations and accounts for random effects), which strengthens the validity and reliability of the observed results as compared to hierarchical regression analyses^17^ and generalized estimating equations^22,23^.

Our findings are in line with the predictions of the neurovisceral integration model^10^, which proposes that autonomic dysregulation, and impaired vagal functioning in particular, is a key pathway linking chronic stress to exhaustion symptoms^41^. Our finding of enhanced vmHRV causally predicting reduced exhaustion symptoms adds important further insights to the validity of the model, as it provides empirical evidence for the theoretical assumption that ANS balance constitutes a relevant psychophysiological resource, which could dampen the effects of prolonged or recurrent stress on the organism’s health^42^. VmHRV has been suggested as an indicator of brain–heart communication^10^, making reductions in vmHRV a relevant biological pathway through which prolonged or recurrent stress leads to organism exhaustion and eventual disease^10^.

Future studies are needed that focus on refining our understanding of potential moderators and mediators within this proposed model. For instance, we have shown that chronic stress and burnout symptoms alter the appraisal of environmental stressors^43,44^ revealing the plausibility of positive feedback loops within the proposed model which deserve further examination. In addition, longitudinal designs on possible pathways from exhaustion symptoms caused by PNS dysregulation to metabolic risk factors and later health outcomes (i.e., cardiovascular disease, diabetes and obesity) are needed, as they could shed light on the mechanisms behind the well-established associations between exhaustion symptoms and the leading cause of death worldwide: cardiovascular diseases^45^.

The finding of a physiological process preceding psychological symptoms suggests its potential usage as a diagnostic biomarker. A diagnostic biomarker can be understood as early detectable biological changes that are indicative of a particular pathogenic process^46^. Diagnostic biomarkers are of special relevance for psychiatry, as they could improve precise diagnoses, which are generally based solely on behavioural symptoms and signs^47^. In addition, biomarkers could signal the pending development of a disorder before major symptoms emerge, potentially lengthening the window of time for prevention^47^. Besides its temporal precedence, a certain specificity of an indicator of a physiological process for the respective psychological symptom has to be proven before it could be used as a diagnostic biomarker. In general, vmHRV has been associated with a wide range of psychopathological syndromes leading to its characterization as a *transdiagnostic biomarker* of psychopathology^48^. While this conceptualization challenges the usage of vmHRV as a specific biomarker for exhaustion symptoms, exhaustion could be conceptualized as a *transdiagnostic symptom* itself, as it constitutes a core element of very different psychopathologies (e.g., present in burnout, depression, chronic fatigue). In such a case, the required specificity of vmHRV would not have to be proven for a certain diagnostic category, but rather for a certain symptom.

Our present findings confirm the predominant role of exhaustion symptoms for the association with vmHRV with respect to other burnout dimensions and depressive symptoms. Correspondingly, neither cynicism nor reduced personal accomplishment was predicted by vmHRV. This is consistent with our previous work^16^, as well as previous research demonstrating that associations with vmHRV were especially strong when burnout measures with a focus on exhaustion were employed^22,23^. From an aetiological standpoint these observations suggest potential differences in the pathogenic mechanisms of the three burnout dimensions. From a methodological point of view, the present findings further challenge the validity of the common practice of combining the three burnout dimensions into a single variable, an approach which has previously been criticized^49^. Our findings indicate that combining these very heterogeneous constructs in one sum score might mask important associations with psychophysiological, and potentially, clinically-relevant variables.

The question regarding whether or not burnout and depressive symptoms are distinct entities is one of the most debated topics since the introduction of the modern burnout concept in the 1970s (review^50^). Undeniably, burnout and depression share chronic stress as a common etiological factor, as well as overlap in symptoms. However, previously made claims of a complete isomorphism of the two concepts is challenged by differences in their symptomatic core features. Depression is characterized by depressed mood, anhedonia, and feelings of hopelessness and guilt^30^ whereas the core components of burnout are, following the definition of the World Health Organization within the International Classification of Diseases (11^th^ Revision^8^), exhaustion, cynicism and reduced personal accomplishment. Moreover, factor analytic studies demonstrated that burnout symptoms can be psychometrically distinguished from depressive symptoms^51,52^.

Our findings offer insights to this debate, by supporting preliminary evidence that biological differences might help to characterize overlap and differences between the two concepts^53^. In contrast to the conclusion of recent arguments that there is no conclusive evidence that differences in HRV are able to differentiate burnout and depressive symptoms^53^ we could show that exhaustion symptoms are predicted by vmHRV even with depressive symptoms included as a covariate. Additionally, in separate analyses depressive symptoms were not predicted by vmHRV when exhaustion symptoms were covaried. These findings contradict prior arguments that relations between burnout symptoms and ANS functioning are mainly driven by depressive symptoms. In contrast, our results suggest that vmHRV is neither a burnout nor depression specific marker but instead is indicative of exhaustion.

Further clarification of the role of exhaustion in linking and/or distinguishing burnout and depressive symptoms is essential, especially with respect to (pharmacological) treatment. On the one hand, there have been warnings that the usage of labels (i.e., burnout, exhaustion), which have not been empirically validated, increases the risk of under- or inappropriate diagnosis and treatment of depressive episodes^50,54^ with potentially severe consequences (i.e., increased risk of suicide). On the other hand, treating exhaustion symptoms with therapeutic approaches common to depression^55^, namely antidepressants might also have serious health consequences. Keeping in mind our finding of significantly reduced vmHRV in individuals with exhaustion symptoms, as well as the predictive value of a hypoactive vagus for CVD risk and mortality^56^ the findings of a meta-analysis that especially tricyclic antidepressants (TCA; e.g., amitriptyline, imipramine, and nortriptyline) significantly reduced parasympathetic tone presumably because of anticholinergic effects^26^, indicates that administering antidepressants to individuals with mainly exhaustion symptoms may pose a considerable risk to the cardiovascular system. Based on these findings, health care providers should be cautious with both, the re-labelling of depression, and the prescription of TCA for individuals with a predominant exhaustion symptomatology independent of the respective diagnostic framework (i.e., burnout, depression), until methodologically-sound prospective large-scale studies have clarified the role of exhaustion symptoms for the conceptual overlap of burnout and depressive symptoms.

There are some limitations to the present research. First, we employed only self-report measures to assess psychological and health-related factors. Therefore, in the present study burnout and depression are not clinically confirmed diagnoses, but reflect psychometrically-determined symptom scores. In our sample, the mean values of burnout and depressive symptoms were higher than in other studies that relied on more or less representative samples^57,58^. Therefore, in addition to general problems of self- report measures (e.g., social desirability^59^), further measurement bias has to be considered, as variability in the presence and intensity of current depressive and burnout symptoms might have influenced the frequency of endorsing particular items. Second, even though we adjusted for a wide range of potential confounding variables we cannot rule out that other variables and conditions (e.g., previous illnesses, physical activity, medication intake, measurement timing) might have also influenced the revealed effects. Our additionally conducted sensitivity analyses further underline the importance of considering additional covariates, whereby larger samples are necessary for this. With our sample size, it seems unclear whether the deviations found between main analyses and sensitivity analyses reflect actual effects of these covariates or are the result of too little power due to the inclusion of too many covariates relative to the sample size. Third, the indicative power of our effects are limited to basal vagal tone, as vmHRV was recorded only during a resting state. We and others have focused primarily on resting state vmHRV as previous longitudinal research clearly supports the reliability of short basal/resting measures (r ≈ 0.60)^60,61^ whereas stress-related HRV has been shown to exhibit modest to low temporal stability (i.e., r = 0.20–30)^62^.

Our study is also characterized by several strengths. Notably, we employed a robust analytic methodology which has several important advantages compared to traditional multiple regression techniques, foremost by maximising the utility of our large sample size by avoiding its reduction due to missing data at single time points. In addition, ours is the first study relating exhaustion and vagal function to cover such an extensive observation period. As such, the present results extend and clarify our and other previous observations indicating the importance of prospective studies with a sufficiently long measurement period. Lastly, by analysing RMSSD and HF-HRV in parallel, we were able to provide an internal replication of the results, which further supports the robustness of the revealed effects.

In conclusion, this is the first study to provide evidence that resting vmHRV is predictive of exhaustion symptoms up to a three-year period in a large, heterogeneous sample. Although replication and additional research are needed, our findings may have crucial implications in the context of growing evidence that increased exhaustion symptoms are a long-term consequence of SARS-CoV-2 (severe acute respiratory syndrome coronavirus type 2) infections^63^, as well as the astonishing increase in chronic work stress, exhaustion and burnout among healthcare providers and others frontline workers during the SARS-CoV-2 pandemic^64,65^. Notably, previous research has shown that hypoactive vagal function is modifiable through smoking cessation^66^, increasing physical activity^67^, and reducing obesity^68^. Thus, our finding of enhanced vmHRV causally predicting reductions in exhaustion symptoms underlines the potential utility of such approaches in exhaustion prevention and treatment.

## Supplementary Information

Supplementary Tables.

## Data Availability

The data that support the findings of this study are available from the corresponding author, [M.K.W.], upon reasonable request.
