# Supplementary material for: Determining the direction of prediction of the association between parasympathetic dysregulation and exhaustion symptoms
Source: Sci Rep. 2022 Jun 23;12:10648. doi: 10.1038/s41598-022-14743-4 (PMC9219378; doi:10.1038/s41598-022-14743-4)
Supplement: Supplementary file 1 — Supplementary Tables. [file 41598_2022_14743_MOESM1_ESM.pdf]

Appendix

Sensitivity analyses including cardiovascular disease (i.e., hypertension, cardiac arrhythmias) and cardiovascular risk factors (i.e., diabetes, high cholesterol) within the multilevel cross-lagged panel models of the main analyses (supplementary tables 1-4).

Supplementary table 1 Path coefficients of cross-lagged panel models on exhaustion symptoms and lnRMSSD with covariates

	lnRMSSD- Exhaustion model			
	lnRMSSD		Exhaustion	
	Estimate	CI	Estimate	CI
Stability paths (T1-T2)	0.57*	0.50, 0.64	0.47*	0.41, 0.52
Cross-lagged effects (T1-T2)	Exhaustion → lnRMSSD		lnRMSSD → Exhaustion	
	-0.03*	-0.06, -0.00	-0.15*	-0.28, -0.02
Effects of covariates (T1)				
Sex	0.02	-0.06, 0.10	-0.01	-0.16, 0.14
Age	-0.01*	-0.01, -0.01	<0.01	-0.01, 0.01
BMI	<0.01	-0.01, 0.00	<0.01	-0.02, 0.01
PHQ-9 som	0.02	-0.00, 0.04	0.11*	0.07, 0.14
PHQ-9 cog	-0.02	-0.04, 0.00	0.15*	0.11, 0.18
Smoking	-0.14*	-0.24, -0.03	0.20	-0.00, 0.41
Alcohol consmption	<0.01	-0.12, 0.12	0.09	-0.14, 0.32
Hypertension	0.02	-0.09, 0.12	-0.11	-0.33, 0.10
Cardiac arrhythmia	0.08	-0.14, 0.03	0.11	-0.32, 0.54
Diabetes	-0.11	-0.31, 0.08	0.32	-0.06, 0.70
High cholesterol	0.02	.0.10, 0.14	-0.02	-0.25, 0.21

Note. Sex is coded 0 = male, 1 = female; BMI = body mass index; CI = confidence interval; lnRMSSD = root mean square of successive difference between heart beats, logarithmized; PHQ-9 cog = Patient Health Questionnaire 9 - cognitive factor; PHQ-9 som = Patient Health Questionnaire 9 - somatic factor; T1 = first attended measurement time point; T2 = measurement time point with the largest time-lag to T1. * CI does not include zero.

Supplementary table 2 Path coefficients of cross-lagged panel models on exhaustion symptoms and lnHF-HRV with covariates

	lnHF-HRV- Exhaustion model			
	lnHF-HRV		Exhaustion	
	Estimate	CI	Estimate	CI
Stability paths (T1-T2)	0.55*	0.49, 0.62	0.47*	0.41, 0.53
Cross-lagged effects	Exhaustion → lnHF-HRV		lnHF-HRV → Exhaustion	
(T1-T2)	-0.06	-0.12, 0.00	-0.06	-0.12, 0.01
Effects of covariates (T1)				
Sex	0.19*	0.02, 0.35	-0.01	-0.16, 0.15
Age	-0.02*	-0.03, -0.01	<0.01	-0.00, 0.01
BMI	-0.01	-0.02, 0.01	<0.01	-0.02, 0.01
PHQ-9 som	0.02	-0.02, 0.07	0.10*	0.06, 0.14
PHQ-9 cog	-0.04*	-0.08, -0.00	0.15*	0.11, 0.19
Smoking	-0.22	-0.45, 0.00	0.21*	0.00, 0.41
Alcohol consumption	-0.05	-0.29, 0.20	0.09	-0.14, 0.32
Hypertension	0.01	-0.22, 0.25	-0.10	-0.30, 0.11
Cardiac arrhythmia	0.01	-0.45, 0.48	0.12	-0.31, 0.56
Diabetes	-0.19	-0.63, 0.23	0.34	-0.05, 0.73
High cholesterol	0.05	-0.21, 0.31	-0.01	-0.25, 0.23

Note. Sex is coded 0 = male, 1 = female; BMI = body mass index; CI = confidence interval; lnHF-HRV = high frequency heart rate variability, logarithmized; PHQ-9 cog = Patient Health Questionnaire 9 - cognitive factor; PHQ-9 som = Patient Health Questionnaire 9 - somatic factor; T1 = first attended measurement time point; T2 = measurement time point with the largest time-lag to T1. * CI does not include zero.

Supplementary table 3 path coefficients of cross-lagged panel models on burnout scores and lnRMSSD with covariates

	**lnRMSSD-MBI model**				**lnRMSSD-CY model**				**lnRMSSD-PEr model**			
	lnRMSSD		MBI		lnRMSSD		CY		lnRMSSD		Per	
	Estimate	CI	Estimate	CI	Estimate	CI	Estimate	CI	Estimate	CI	Estimate	CI
Stability paths (T1-T2)	0.58*	0.51, 0.65	0.52*	0.46, 0.57	0.58*	0.51, 0.65	0.50*	0.44, 0.56	0.58*	0.51, 0.65	0.51*	0.45, 0.58
Cross-lagged effects (T1-T2)	MBI → lnRMSSD		lnRMSSD → MBI		CY → lnRMSSD		lnRMSSD → CY		Per→ lnRMSSD		lnRMSSD → Per	
	-0.01	-0.05, 0.03	-0.07	-0.16, 0.01	0.01	-0.01, 0.04	-0.10	-0.26, 0.06	0.02	-0.02, 0.06	0.03	-0.08, 0.14
Effects of covariates (T1)												
Sex	0.01	-0.07, 0.09	-0.04	-0.14, 0.06	0.01	-0.07, 0.09	-0.10	-0.27, 0.07	0.01	-0.07, 0.09	-0.01	-0.14, 0.12
Age	-0.01*	-0.01, -0.01	<0.01	-0.00, 0.01	-0.01*	-0.01, -0.01	<0.01	-0.01, 0.01	-0.01*	-0.01, -0.01	<0.01	-0.00, 0.01
BMI	-0.01	-0.01, 0.00	<0.01	-0.01, 0.01	-0.01	-0.01, 0.00	0.02*	0.00, 0.04	<0.01	-0.01, 0.01	-0.01	-0.02, 0.01
PHQ-9 som	0.02	-0.01, 0.04	0.04*	0.02, 0.07	0.01	-0.01, 0.03	0.02	-0.03, 0.06	0.01	-0.01, 0.03	<0.01	-0.03, 0.03
PHQ-9 cog	-0.02*	-0.04, -0.00	0.13*	0.11, 0.16	-0.03*	-0.05, -0.01	0.16*	0.12, 0.21	-0.03*	-0.05, -0.01	0.09*	0.06, 0.12
Smoking	-0.14*	-0.24, -0.03	0.13	-0.01, 0.27	-0.14*	-0.24, -0.04	0.10	-0.14, 0.34	-0.14*	-0.24, -0.04	0.07	-0.10, 0.24
Alcohol consmuption	<0.01	-0.11, 0.12	0.07	-0.09, 0.23	<0.01	-0.11, 0.12	0.11	-0.16, 0.38	<0.01	-0.12, 0.13	<0.01	-0.18, 0.19
Hypertension	0.02	-0.08, 0.13	-0.05	-0.19, 0.09	0.03	-0.08, 0.14	-0.13	-0.39, 0.13	0.03	-0.08, 0.14	0.09	-0.09, 0.27
Cardiac arrhythmia	0.07	-0.16, 0.29	0.19	-0.11, 0.50	0.05	-0.18, 0.28	0.42	-0.08, 0.93	0.07	-0.14, 0.29	0.09	-0.26, 0.44
Diabetes	-0.12	-0.31, 0.08	0.08	-0.19, 0.34	-0.11	-0.32, 0.09	-0.09	-0.53, 0.37	-0.12	-0.32, 0.07	-0.07	-0.38, 0.25
High cholesterol	0.03	-0.09, 0.15	-0.06	-0.21, 0.11	0.04	-0.08, 0.15	-0.19	-0.47, 0.08	0.03	-0.09, 0.16	<0.01	-0.19, 0.20

Note. Sex is coded 0 = male, 1 = female; BMI = body mass index; CI = confidence interval; CY = Maslach Burnout Inventory – cynicism sub score; lnRMSSD = root mean square of successive difference between heart beats, logarithmized; MBI = Maslach Burnout Inventory GS – total score; Per = Maslach Burnout Inventory – reduced personal accomplishment sub score; PHQ-9 cog = Patient Health Questionnaire 9 - cognitive factor; PHQ-9 som = Patient Health Questionnaire 9 - somatic factor; T1 = first attended measurement time point; T2 = measurement time point with the largest time-lag to T1. * CI does not include zero.

Supplementary table 4 Path coefficients of cross-lagged panel models on PHQ-9 factors and lnRMSSD with covariates

	**lnRMSSD-PHQ-9 som model**				**lnRMSSD-PHQ-9 cog model**			
	lnRMSSD		PHQ-9 som		lnRMSSD		PHQ-9 cog	
	Estimate	CI	Estimate	CI	Estimate	CI	Estimate	CI
Stability paths (T1-T2)	0.58*	0.51, 0.56	0.61*	0.56, 0.67	0.58*	0.51, 0.65	0.67*	0.62, 0.73
Cross-lagged effects (T1-T2)	PHQ-9 som → lnRMSSD		lnRMSSD → PHQ-9 som		PHQ-9 cog → lnRMSSD		lnRMSSD → PHQ-9 cog	
	-0.01	-0.02, 0.01	-0.20	-0.49, 0.08	-0.01	-0.02, 0.01	-0.03	-0.33, 0.26
Effects of covariates (T1)								
Sex	0.03	-0.05, 0.10	0.10	-0.24, 0.44	0.02	-0.06, 0.10	-0.11	-0.45, 0.25
Age	-0.01*	-0.01, -0.01	-0.01	-0.03, 0.00	-0.01*	-0.01, -0.01	-0.02*	-0.04, -0.00
BMI	<0.01	-0.01, 0.00	0.03	-0.01, 0.07	<0.01	-0.01, 0.00	0.03	-0.01, 0.07
Smoking	-0.15*	-0.25, -0.05	-0.01	-0.46, 0.44	-0.15*	-0.25, -0.04	0.24	-0.23, 0.68
Alcohol consumption	<0.01	-0.12, 0.12	-0.56*	-1.08, -0.03	<0.01	-0.12, 0.12	-0.13	-0.67, 0.42
Hypertension	0.02	-0.09, 0.13	-0.41	-0.87, 0.06	0.02	-0.09, 0.13	-0.11	-0.59, 0.38
Cardiac arrhythmia	0.07	-0.16, 0.29	-0.85	-0.07, 1.82	0.07	-0.15, 0.29	0.42	-0.58, 1.42
Diabetes	-0.12	-0.31, 0.08	0.04	-0.85, 0.92	-0.12	-0.33, 0.08	-0.08	-0.97, 0.81
High hholesterol	0.03	-0.08, 0.15	0.40	-0.11, 0.91	0.03	-0.09, 0.16	0.22	-0.30, 0.74

Note. Sex is coded 0 = male, 1 = female; BMI = body mass index; CI = confidence interval; lnRMSSD = root mean square of successive difference between heart beats, logarithmized; PHQ-9 cog = Patient Health Questionnaire 9 - cognitive factor; PHQ-9 som = Patient Health Questionnaire 9 - somatic factor; T1 = first attended measurement time point; T2 = measurement time point with the largest time-lag to T1. * CI does not include zero.
